# The Scientific American

**Published:** 1880-12

**Authors:** 


*The Scientific American.*—

This useful and interesting publication will enter upon the 37th year of its existence in January, 1881. All classes of men seek its pages for information, suggestion. or entertainment, and having no rival, its editors and publishers are certainly striving to increase the usefulness of the paper to its readers and advertisers, as an examination of the volume just finished will clearly indicate. Every volume published has contained a large amount of original matter of special value to scientific and practical men, together with the best contributions to the practical literature of the sciences and industrial arts that the public journals afford. The *Scientific American* and Supplement are both valuable papers, the former at a subscription price of $3.20 per year, issued in weekly numbers; the latter also weekly at $5.00 per year, or the two published at $7.00 per year. Munn & Co., Editors and Proprietors, No. 37 Park Row, New York.

